# Asthma prescribing, ethnicity and risk of hospital admission: an analysis of 35,864 linked primary and secondary care records in East London

**DOI:** 10.1038/npjpcrm.2016.49

**Published:** 2016-08-18

**Authors:** Sally A Hull, Shauna McKibben, Kate Homer, Stephanie JC Taylor, Katy Pike, Chris Griffiths

**Affiliations:** 1 Clinical Effectiveness Group, Centre for Primary Care and Public Health, Queen Mary University of London, London, UK; 2 Asthma UK Centre for Applied Research, Queen Mary University of London, London, UK; 3 Respiratory Critical Care and Anaesthesia Section, University College London, Institute of Child Health, London, UK

## Abstract

Inappropriate prescribing in primary care was implicated in nearly half of asthma deaths reviewed in the UK’s recent National Review of Asthma Deaths. Using anonymised EMIS-Web data for 139 ethnically diverse general practices (total population 942,511) extracted from the North and East London Commissioning Support Unit, which holds hospital Secondary Uses Services (SUS)–linked data, we examined the prevalence of over-prescribing of short-acting β_2_-agonist inhalers (SABA), under-prescribing of inhaled corticosteroid (ICS) inhalers and solo prescribing of long-acting β_2_-agonists (LABA) to assess the risk of hospitalisation for people with asthma for 1 year ending August 2015. In a total asthma population of 35,864, multivariate analyses in adults showed that the risk of admission increased with greater prescription of SABA inhalers above a baseline of 1–3 (4–12 SABA: odds ratio (OR) 1.71; 95% confidence interval (CI) 1.20–2.46, ⩾13 SABA: OR 3.22; 95% CI 2.04–5.07) with increasing British Thoracic Society step (Step 3: OR 2.90; 95% CI 1.79–4.69, Step 4/5: OR 9.42; 95% CI 5.27–16.84), and among Black (OR 2.30; 95% CI 1.64–3.23) and south Asian adult populations (OR 1.83; 95% CI 1.36–2.47). Results in children were similar, but risk of hospitalisation was not related to ethnic group. There is a progressive risk of hospital admission associated with the prescription of more than three SABA inhalers a year. Adults (but not children) from Black and South Asian groups are at an increased risk of admission. Further work is needed to target care for these at-risk groups.

## Introduction

Frequent use of short-acting β_2_-agonist (SABA) inhaler medication is a key indicator of poor asthma control,^1,2^ a risk factor for asthma exacerbations^3^ and a potentially modifiable risk factor for asthma-related death.^4,5^ Clinical guidelines recommend that those using more than one SABA canister (200 actuations) per month should have an early clinical review.^5,6^ The UK National Review of Asthma Deaths (NRAD)^4^ identified poor prescribing practice and a failure by clinicians to adhere to British Thoracic Society (BTS)/SIGN asthma guidelines. In 39% of the 195 asthma deaths reviewed, >12 SABA inhalers had been prescribed in the previous year; >50 SABA inhalers had been prescribed in 4% of cases; and in 3% of cases long-acting β_2_-agonist (LABA) monotherapy was prescribed without inhaled corticosteroid (ICS) preventer treatment.^4^

Asthma morbidity disproportionately affects people from ethnic minority backgrounds.^7^ Often concentrated in inner-city areas,^8,9^ ethnic minorities often have poorer continuity with healthcare providers,^10^ less familiarity with primary healthcare practitioners^11^ and are more likely to present to Accident and Emergency Departments (AED) as a primary source of care.^12^ In 1998, UK hospitalisation rates for asthma in adults and children of black and south Asian origin were found to be higher than in the white British population.^13^ Recent data from Scotland showed an increased risk of hospitalisation for Pakistani and Indian adults^14^ but did not provide analyses for ethnic minority children. Figures for UK emergency asthma admission rates showed that the East London boroughs included in our study all had crude admission rates above the average for London (1.02/1000 population) in 2012–13.^15^

Studies suggest that differences in asthma medication use may contribute to asthma morbidity in ethnic minority populations,^16,17^ yet these groups are often under-represented in asthma research.

Inappropriate use of asthma medications has been reported as a major contributor to uncontrolled asthma in inner-city populations,^18,19^ particularly in children from inner-city areas who are more likely to overuse SABA on a daily basis^18^ and are the least likely to receive adequate guideline-based therapy^17,20^ or specialty asthma care.^21–23^ In US research by Crocker *et al.*^18^ and Butz *et al.,*^24^ SABA use was high among inner-city children, with hospitalisation for asthma five times more likely in high SABA users than in low-to-moderate users,^24^ whereas twice as many black children had asthma-related AED visits (39 vs 18%) and hospitalisations (12 vs 5%,) compared with white children.^18^

To examine the prevalence of sub-optimal prescribing and differences in asthma outcomes, this study linked primary and secondary care records from a large multi-ethnic inner-city population of patients aged 5–75 years with asthma, to address the following questions:

- How common is the excessive prescription of SABA inhalers for people with asthma?
- How common is the prescription of LABA inhalers without co-prescription of an ICS?
- Is excessive SABA prescribing and solo LABA prescribing associated with adverse morbidity, including hospitalisation?
- Which population groups are at an increased risk of hospitalisation with asthma?

## Results

Among our study population of 35,864 cases, 67% of the child and adolescent groups and 46% of the adult groups were of Black or South Asian ethnicity (Table 1). Smoking rates for adults with asthma (20%) are similar to that for the whole East London population.^25^ Over 93% of patients had an asthma review recorded and 82% had an asthma management plan. BTS asthma treatment step was recorded for 86%. The overall annual non-elective admission episode rate for asthma was 2.4/100 population. Crude admission episode rates were highest among the youngest group (Table 1).

### Excess SABA prescribing

Among the total asthma population, 10.2% (3,671/35,864) were prescribed >12 SABA inhalers a year (Table 1). These high rates of SABA prescribing were observed among 5.9% of children and adolescents, and in 13.6% of adults.

### Treatment by SABA alone

Table 2 shows asthma severity, primary care management and hospital admissions among the 8,227 cases (22.8%) in whom SABA inhalers were prescribed as the sole treatment. BTS asthma treatment step 1 was recorded in 68% of cases, and in 87% of cases an asthma review was carried out in the previous year. Within this group, 3.5% (285 cases) were prescribed >12 SABA inhalers a year. The crude asthma admission rate was 0.67%.

### Treatment by ICS or COMBI with SABA

The majority (27,637, 77%) of the population with asthma was prescribed ICS or COMBI treatment, as well as SABA inhalers. Among these, 20,884 (76%) were prescribed fewer than 10 ICS canisters a year, which we considered a marker of sub-optimal preventer treatment.^4^

Table 3 shows asthma severity, primary care management and hospital admissions among the 20,884 cases who were prescribed <10 ICS or combination inhalers—ICS and Long-acting β_2_-agonist (COMBI) inhalers a year. Within this group, BTS asthma treatment step 2 was recorded in 65%, and in 95% of cases an asthma review was carried out in the previous year; 5.5% of cases were prescribed >12 SABA canisters a year. The crude hospital admission episode rate for asthma rises from 1.3 to 7.5 per 100 population as the number of SABA inhalers prescribed rises from 1–3 to 13+ a year.

The greatest number of emergency hospital admissions for asthma (447 admissions) occurs in this group of people prescribed <10 ICS inhalers a year. Among this group, we estimate a number needed to treat (NNT) of 16 to potentially reduce the 7.5% risk of admission in the high SABA use group to the 2% risk of admission seen in those using ⩽12 SABA inhalers.

### Use of LABA without concomitant inhaled steroids

Among the asthma population (excluding all those with a read coded COPD diagnosis), 0.3% (105) of the cases had a LABA prescribed without ICS. Asthma reviews were completed for 88% of cases, and there were no admissions among this group (Supplementary Appendix 1).

### Risk of emergency hospital admission

Multivariate analyses (Table 4) showed that in both children and adults the risk of admission rose steadily when SABA prescribing exceeded 1–3 inhalers a year, and when BTS asthma treatment step was >2. Children and women had a higher risk of admission. Among adults, but not children, the risk of admission was increased for those with Black or south Asian ethnicity in comparison with White groups. Further analysis by ethnicity did not suggest differences in primary care management or prescribing to account for these findings (Table 5).

Supplementary Appendix 2 details the characteristics of the 410 individuals who accounted for 872 episodes of asthma admission. These reflect the factors identified in the multivariate model, and the proportions of asthma reviews and management plans did not differ from the whole asthma population.

## Discussion

### Main findings

We report the first UK population–based analysis of prescribing and risk of hospitalisation in a multi-ethnic population. Among the study population of 35,864 people with asthma, the results showed high levels of recorded asthma reviews (93%) compared with the Quality and Outcomes Framework (QOF) data for England 2014–15, which report that 76% of patients on asthma registers received an asthma review in the previous 12 months.^26^ Our results also showed that 82% of the population received asthma management plans, suggesting a high level of guideline-based primary care within the East London asthma population.

It is chastening to observe that among adults with asthma the prevalence of smoking is the same as the general population. This suggests that although smoking status for those with asthma is well-recorded, smoking cessation interventions in primary care are either under-utilised or insufficient to support people with asthma to stop smoking.

### Prescribing for asthma

Of those prescribed SABA as the sole treatment, 3.5% of cases were prescribed >12 SABA in the previous year. In all, 95% of cases were documented as BTS asthma treatment step 1 or 2, suggesting that those treated with SABA alone had milder asthma and are at a lower risk for admission. However, it is likely that some cases in this group would benefit from additional ICS treatment.

The greatest burden of emergency hospital admission for asthma (447 admissions) occurred among the population requiring inhaled steroids but prescribed less than 10 ICS inhalers a year. In this group, rising numbers of prescribed SABA canisters is a clear marker of risk for hospital admission.

In a small number of cases (0.3%), LABAs were prescribed without an ICS. LABAs have the potential to increase the risk of asthma mortality when used by patients with unstable asthma without concomitant ICS therapy or scheduled medical review;^27^ however, there is no evidence of an increased risk of asthma mortality with combination ICS/LABA inhaler therapy in asthma.^28^

### Interpretation of findings in relation to previously published work

Reports from North America suggest that increasing use of more than three SABA inhalers per year is associated with an increased risk of asthma-related hospitalisation.^1,24,29–33^ In the absence of similar studies in the UK, our results confirm that using more than three SABA inhalers per year is an independent risk factor for asthma-related hospitalisation in both children and adults.

Previous studies^12,18,34^ have found that children from ethnic minority groups have higher rates of admission than those in the White population, with the most recent UK data reported in 1998.^13^ We did not observe this difference, suggesting that all children in East London get equal access to primary asthma care management. A recent report on ethnic variations in asthma hospital admission, re-admission and deaths in Scotland^14^ observed a modestly increased rate of hospital admission for asthma among the Pakistani and Indian populations, but not in those of African ethnicity. In contrast, we find that adults from Black or south Asian ethnicity have a twofold risk of admission in comparison with those of White background. We find no indication that these groups have more severe asthma, or have different patterns of primary care management or prescribing. This suggests that there may be differences in health-seeking behaviour or self-management related to culture, language or asthma understanding, which we are not able to capture in a database study.

### Strengths and limitations of this study'

These results are based on asthma cases from a population of almost one million GP-registered patients in East London, linked to ‘real-time’ hospital activity data. The advantage of this contiguous practice population data set is that it avoids the selective bias of practice ‘opt-in’. Results from this study will be generalisable to other inner urban populations in the UK. With over 93% ethnicity recording in this urban, multi-ethnic population, we are able to explore the contribution of ethnicity and deprivation alongside other established risk factors for asthma admission.

The prescribing data are based on electronic prescriptions issued by GPs; hence, this will include non-dispensed items (lost, unused, incorrect) in contrast to studies elsewhere, which have used dispensing data.^1,24,29–32^ In the absence of medication-dispensing data linked with medical records in the UK, patient-level primary care prescribing data can be used to assess medication usage^35^ and is a useful predictor of dispensing-based adherence.^36^ In addition, we were not able to link oral steroid use for asthma exacerbations, or capture ‘rescue steroid courses’ initiated by patients.

Although we obtained Secondary Uses Services (SUS) data on asthma-related hospitalisations, we were unable to examine asthma attendances at AED because of the low rates of AED problem coding in the linked data set (see Materials and methods section).

### Implications for future research, policy and practice

The electronic surveillance of prescription refill frequency in primary care has been recommended to alert clinicians to patients being prescribed excessive quantities of SABAs^4^ to allow for more frequent monitoring of those at risk of exacerbation. Computer decision support systems, which include electronic alerts, are increasingly used globally in the policy drive to improve prescribing safety. Those with asthma who are under-prescribed ICS and over-prescribed SABA account for the majority of hospital admissions. We recommend that electronic surveillance of prescribing be introduced in primary care practices to identify this at-risk population. Previous research shows that disproportionately few patients are responsible for a large component of total asthma direct medical costs;^33^ therefore, the identification of high-risk asthma populations might allow for targeted interventions to improve asthma control and reduce healthcare resource use.

Despite guideline recommendations that LABA should be prescribed in fixed-dose-combination ICS/LABA inhalers for the treatment of asthma,^5,6^ our results show that clinicians do not always adhere to guidelines. We support previous recommendations^37^ suggesting practice strategies which detect and feedback to clinicians inappropriate LABA monotherapy. Furthermore, LABA/ICS therapy should only be available for prescription in a single device combination inhaler for the management of asthma.

### Conclusions

In this study, we identified a twofold risk in hospital admissions for asthma in adults of ethnic minority backgrounds but not in children and adolescents of ethnic minority backgrounds. We found no variation in the prescribing and management of ethnic minority populations to account for these differences. A variety of possible explanations have been suggested for the observed ethnic inequalities in the burden of asthma,^38^ including lifestyle and environment, culture, communication issues and the nature of doctor–patient interactions. Further research addressing the social, cultural and psychological issues experienced by ethnic minorities is necessary to explain ethnic variations in asthma hospitalisations and to effect change in asthma outcomes.

## Materials and methods

This study was set in the three geographically contiguous East London clinical commissioning groups (CCGs) of Newham, Tower Hamlets and City and Hackney, with a combined GP-registered population of 942,511 in 2015. In the 2011 UK census, 48% of the population in these three CCGs was recorded to be of non-White ethnic origin and among the eight most socially deprived localities in Britain.^39^

### Data sources

In August 2015, anonymised demographic and clinical data were extracted from the North and East London Commissioning Support Unit (NELCSU), which holds EMIS-Web primary care data linked to hospital SUS data.^40^ Data were obtained for all patients aged 5–75 years: children (5–11 years), adolescents (12–17 years), adults (18–54 years) and mature adults (55–75 years) currently registered with 139/140 general practices in the area. Data were extracted on secure N3 terminals using SQL Server Management Studio (2014), which interfaces with the CSU databases. All data were anonymous and managed according to UK NHS information governance requirements.

### Clinical and prescribing variables

The study population was identified using the read code set for asthma used by the UK QOF. Among 942,511 registered patients, we identified 35,864 cases, aged 5–75 years, with a recorded diagnosis of asthma, and who were prescribed ⩾1 inhaled asthma medication in the previous 12 months. This formed our study population for analysis.

We extracted data on clinical variables, including smoking status, influenza immunisation and the presence of COPD, and aspects of asthma care, including the BTS asthma treatment steps, the presence of asthma review, inhaler technique and asthma management plans. For all asthma care variables, the most recent value in the previous year (August 2014–August 2015) was used. Prescribing data included all inhaled asthma medications issued in the previous year. Data were converted to annual numbers of inhalers issued in the following therapeutic categories, SABA, LABA, ICS and COMBI.

### Ethnicity and demographic data

Self-reported ethnicity was recorded at the practice during registration or routine consultation. Ethnic categories are based on the UK 2011 census and for this study were condensed into three major categories: White (British, Irish, other White), Black (Black African, Black Caribbean, Black British, other Black and mixed Black) and South Asian (Bangladeshi, Pakistani, Indian, Sri Lankan, British Asian, other South Asian or mixed Asian). The index of multiple deprivation (IMD2010) score (derived from census variables and linked to patient place of residence) was used as a measure of social deprivation, categorised in quintiles of the study population.^41^

Health Service use Primary care consultation data for the previous year were extracted as contacts with General Practitioners either in the surgery or at home, and telephone contacts. Non-elective inpatient hospital admissions where asthma was recorded as the primary cause were collected using International Classification of Diseases (ICD-10) codes for Asthma (J45) and Acute Severe Asthma (J46).^42^ Data were extracted for all AED visits for the study population in the previous year. However, the reason for AED attendance was missing in >95% of instances; hence, these data were not used in the analysis.

### Statistical analysis

Statistical analyses were performed using Stata version 12 (StataCorp LLP, College Station, TX, USA). On the basis of advice from community pharmacists and CCG-prescribing advisors, inhaler prescriptions were examined, and those patients with more than four inhalers of any one type prescribed on a single date were excluded from the analysis as likely to represent prescribing errors. This constituted 289/36,153 (0.8%) of cases. To examine the contribution of factors to risk of admission with asthma, multivariate models were constructed using potential explanatory variables (Table 1). We examined collinearity, finding a significant association (Spearman’s *ρ*=0.6) between the prescribing rates of SABA and ICS+COMBI. As potentially important clinical predictors of hospital admission, both were included in the final model.

## Figures and Tables

**Table 1:** Characteristics of the study population (*N*=35,864)

	*Total*	*Children*	*Adolescents*	*Adult*	*Mature adults*
Age (years)	All *N* (%)	5–11 *N* (%)	12–17 *N* (%)	18–54 *N* (%)	55–75 *N* (%)
	35,864 (100.0)	3,585 (10.0)	3,269 (9.1)	21,581 (60.2)	7,429 (20.7)
					
*Gender*					
Female	19,113 (53.3)	1,353 (37.7)	1,309 (40.0)	11,891 (55.1)	4,560 (61.4)
					
*Ethnicity*					
White	14,083 (39.3)	650 (18.1)	547 (16.7)	9,423 (43.7)	3,463 (46.6)
South Asian	11,921 (33.2)	1,634 (45.6)	1,554 (47.5)	6,442 (29.9)	2,291 (30.8)
Black	5,925 (16.5)	676 (18.9)	698 (21.4)	3,487 (16.2)	1,064 (14.3)
Other	1,522 (4.2)	161 (4.5)	123 (3.8)	879 (4.1)	359 (4.8)
Unknown^a^	2,413 (6.7)	464 (12.9)	347 (10.6)	1,350 (6.3)	252 (3.4)
					
*Clinical measures*					
Asthma review	33,531 (93.5)	3,203 (89.3)	3,149 (96.3)	19,925 (92.3)	7,254 (97.6)
Asthma management plan	29,352 (81.8)	2,689 (75.0)	2,838 (86.8)	17,246 (79.9)	6,579 (88.6)
Asthma step recorded	30,886 (86.1)	2,817 (78.6)	2,932 (89.7)	18,204 (84.4)	6,933 (93.3)
Inhaler technique checked	30,201 (84.2)	2,548 (71.1)	2,880 (88.1)	17,791 (82.4)	6,982 (94.0)
Flu immunisation^b^	20,458 (57.0)	1,680 (46.9)	1,517 (46.4)	11,251 (52.1)	6,010 (80.9)
Currently smoking	6,188 (17.3)	(0.0)	52 (1.6)	4,938 (22.9)	1,198 (16.1)
COPD diagnosis	1,613 (4.5)	2 (0.1)	3 (0.1)	396 (1.8)	1,212 (16.3)
					
*Asthma severity*^c^					
Mild intermittent asthma—Step 1	7,660 (24.8)	847 (30.1)	975 (33.3)	4,746 (26.1)	1,092 (15.8)
Regular preventer therapy—Step 2	16,863 (54.6)	1,780 (63.2)	1,717 (58.6)	9,812 (53.9)	3,554 (51.3)
Initial add-on therapy—Step 3	5,595 (18.1)	171 (6.1)	221 (7.5)	3,228 (17.7)	1,975 (28.5)
Persistent poor control—Step 4	718 (2.3)	19 (0.7)	18 (0.6)	394 (2.2)	287 (4.1)
Severe asthma—Step 5	50 (0.2)	(0.0)	1 (0.0)	24 (0.1)	25 (0.4)
					
*Healthcare resource use*					
Count GP consultations (median)	158,873 (3)	10,571 (2)	8,428 (2)	93,322 (3)	46,552 (5)
Count IP episodes (median)	872 (0)	131 (0)	55 (0)	498 (0)	188 (0)
Crude IP episode, rate per 100 population	2.43	3.65	1.68	2.31	2.53
					
*SABA inhalers prescribed*					
0	1,709 (4.8)	72 (2.0)	61 (1.9)	1,135 (5.3)	441 (5.9)
1–3	15,611 (43.5)	1,557 (43.4)	1,648 (50.4)	10,408 (48.2)	1,998 (26.9)
4–12	14,873 (41.5)	1,759 (49.1)	1,355 (41.4)	8,125 (37.6)	3,634 (48.9)
13+	3,671 (10.2)	197 (5.5)	205 (6.3)	1,913 (8.9)	1,356 (18.3)
					
*Total ICS+COMBI inhalers prescribed*					
0	8,354 (23.3)	817 (22.8)	915 (28.0)	5,482 (25.4)	1,140 (15.3)
1–9	22,340 (62.3)	2,567 (71.6)	2,134 (65.3)	13,579 (62.9)	4,060 (54.7)
10+	5,170 (14.4)	201 (5.6)	220 (6.7)	2,520 (11.7)	2,229 (30.0)

Abbreviations: COMBI, combination inhalers (ICS and Long-acting β_2_-agonist); COPD, chronic obstructive pulmonary disease; GP, general practitioner; ICS, inhaled corticosteroid; IP, inpatient; SABA, short-acting β_2_-agonist.

a Unknown ethnic group=not stated code or missing.

b Flu immunisation in the past year.

c Asthma step as a proportion of total with asthma step recorded.

**Table 2:** Severity and clinical management of asthma population prescribed SABA alone, by number of SABA inhalers (*N*=8,227)

	*Total* N *(%)*	*1–3* N *(%)*	*4–12* N *(%)*	*13+N (%)*
	8,227 (100.0)	5,888 (71.6)	2,054 (25.0)	285 (3.5)
				
*Clinical measures*				
Asthma review	7,176 (87.2)	4,963 (84.3)	1,936 (94.3)	277 (97.2)
Asthma management plan	5,875 (71.4)	3,977 (67.5)	1,653 (80.5)	245 (86.0)
Asthma step recorded	6,077 (73.9)	4,076 (69.2)	1,740 (84.7)	261 (91.6)
				
*Asthma severity*^a^				
Step 1	4,106 (67.6)	2,901 (71.2)	1,076 (61.8)	129 (49.4)
Step 2	1,683 (27.7)	1,039 (25.5)	543 (31.2)	101 (38.7)
Step 3	270 (4.4)	133 (3.3)	111 (6.4)	26 (10.0)
Step 4/5	18 (0.3)	3 (0.1)	10 (0.6)	5 (1.9)
				
*Healthcare resource use*				
Count IP episodes	55	20	33	2
Crude IP episode, rate per 100 population	0.67	0.34	1.61	0.70

Abbreviations: IP, inpatient; SABA, short-acting β_2_-agonist.

a Asthma step % as a proportion of total with asthma step recorded.

**Table 3:** Severity and clinical management of asthma population prescribed 1–9 ICS+COMBI per year, by number of SABA inhalers (*N*=20,884)

	*Total* N *(%)*	*1–3* N *(%)*	*4–12* N *(%)*	*13+N (%)*
	20,884	9,451 (45.3)	10,285 (49.3)	1,148 (5.5)
				
*Clinical measures*				
Asthma review	19,787 (94.8)	8,739 (92.5)	9,919 (96.4)	1,129 (98.3)
Asthma management plan	17,471 (83.7)	7,603 (80.5)	8,848 (86.0)	1,020 (88.9)
Asthma step recorded	18,498 (88.6)	7,986 (84.5)	9,435 (91.7)	1,077 (93.8)
				
*Asthma severity*^a^				
Step 1	3,060 (16.5)	1,621 (20.3)	1,313 (13.9)	126 (11.7)
Step 2	11,929 (64.5)	5,279 (66.1)	6,085 (64.5)	565 (52.5)
Step 3	3,153 (17.1)	1,023 (12.8)	1,831 (19.4)	299 (27.8)
Step 4/5	356 (1.9)	63 (0.8)	206 (2.2)	87 (8.1)
				
*Healthcare resource use*				
Count IP episodes	447	122	239	86
Crude IP episode, rate per 100 population	2.14	1.29	2.32	7.49

Abbreviations: COMBI, combination inhalers (ICS and Long-acting β_2_-agonist); ICS, inhaled corticosteroid; IP, inpatient; SABA, short-acting β_2_-agonist.

a Asthma step % as a proportion of total with asthma step recorded.

**Table 4:** Multivariate model for risk of admission for children (5–17 years) and for adults (18–75 years)

	*Children (5–17)* N^a^*=6,853*			*Adults (18–75)* N^a^*=28,992*		
	*OR*	*95% CI*	P *value*	*OR*	*95% CI*	P *value*
*Gender*						
Male (ref)	1			1		
Female	1.36	(0.98–1.89)	0.07	1.34	(1.03–1.75)	0.03
						
Age	0.85	(0.81–0.90)	<0.00	0.98	(0.97–0.99)	0.00
						
*Ethnicity*^b^						
White (ref)	1			1		
South Asian	1.47	(0.83–2.62)	0.19	1.83	(1.36–2.47)	<0.00
Black	1.21	(0.61–2.40)	0.58	2.30	(1.64–3.23)	<0.00
						
*Flu immunisation*^c^						
No (ref)	1			1		
Yes	1.01	(0.75–1.38)	0.93	1.03	(0.74–1.43)	0.85
						
*Currently smoking*						
No (ref)	NA			1		
Yes	NA	NA	NA	0.83	(0.61–1.12)	0.23
						
*COPD diagnosis*						
No (ref)	NA			1		
Yes	NA	NA	NA	0.99	(0.59–1.68)	0.98
						
*SABA inhalers prescribed*						
1–3 (ref)	1			1		
0	1.03	(0.25–4.36)	0.96	0.81	(0.37–1.78)	0.60
4–12	1.66	(1.13–2.43)	0.01	1.71	(1.20–2.46)	<0.00
13+	4.09	(2.01–8.34)	<0.00	3.22	(2.04–5.07)	<0.00
						
*Asthma severity*						
Step 1 (ref)	1			1		
Step 2	1.30	(0.76–2.24)	0.33	1.24	(0.79–1.97)	0.35
Step 3	2.73	(1.41–5.28)	<0.00	2.90	(1.79–4.69)	<0.00
Step 4/5	5.54	(1.50–20.51)	0.01	9.42	(5.27–16.84)	<0.00
						
*GP consultation rate per patient*						
0–1 (ref)	1			1		
2–6	1.32	(0.92–1.90)	0.13	1.59	(0.99–2.57)	0.06
>6	2.58	(1.43–4.66)	<0.00	2.80	(1.70–4.63)	0.00

Adjusted for IMD2010 quintile, total ICS+COMBI inhalers and practice clustering.

Abbreviations: CI, confidence Interval; COMBI, combination inhalers (ICS and long-acting β_2_-agonist); COPD, chronic obstructive pulmonary disease; ICS, inhaled corticosteroid; OR, odds ratio; NA, not applicable; SABA, short-acting β_2_-agonist.

a *N*=cases contributing to the model.

b 'Other' and 'unknown' ethnicity categories not shown.

c Flu immunisation in the past year.

**Table 5:** Characteristics of major ethnic groups^a^ for all adults in the asthma population (*N*=29,010), percentages shown

	*Total*^a^*%*	*White %*	*South Asian %*	*Black %*
Number	29,010	12,886	8,733	4,551
				
*Gender*				
Female	56.7	56.9	53.2	63.4
				
*Age bands*				
18–54	74.4	73.1	73.8	76.6
55–75	25.6	26.9	26.2	23.4
				
*Clinical measures*				
Asthma review	93.7	92.4	95.8	95.6
Asthma management plan	82.1	78.9	88.5	84.6
Asthma step recorded	86.6	84.7	90.5	89.0
Inhaler technique checked	85.4	83.1	89.9	89.0
Flu immunisation^b^	59.5	55.0	70.0	56.7
Currently smoking	21.2	27.0	14.5	18.3
COPD diagnosis	5.5	8.6	3.3	2.7
				
*Asthma severity^c^*				
Step 1	23.2	22.8	22.2	24.1
Step 2	53.2	50.1	56.2	56.7
Step 3	20.7	23.4	19.4	17.1
Step 4/5	2.9	3.7	2.2	2.1
				
*SABA inhalers prescribed*				
0	5.4	5.5	5.7	4.6
1–3	42.8	42.1	40.4	45.0
4–12	40.5	40.0	42.2	41.4
13+	11.3	12.4	11.7	9.0
				
*Total ICS+COMBI inhalers prescribed*				
0	22.8	23.7	19.8	22.7
1–9	60.8	59.4	62.2	63.6
10+	16.4	16.9	18.0	13.7
				
*Healthcare resource use*				
Count IP episodes	686	271	246	116
Crude IP episode, rate per 100 population	2.36	2.10	2.82	2.55

Abbreviations: COMBI, combination inhalers (ICS and long-acting β_2_-agonist); COPD, chronic obstructive pulmonary disease; ICS, inhaled corticosteroid; IP, inpatient; SABA, short-acting β_2_-agonist.

a 'Other' and 'unknown' ethnicity categories not shown.

b Flu immunisation in the past year.

c Asthma step % as a proportion of total with asthma step recorded.

## Notes

CG is an Assistant Editor of *npj Primary Care Respiratory Medicine*, but was not involved in the editorial review of, nor the decision to publish this article. The other authors declare no conflict of interest.
