# Supplementary material for: Asthma prescribing, ethnicity and risk of hospital admission: an analysis of 35,864 linked primary and secondary care records in East London
Source: NPJ Prim Care Respir Med. 2016 Aug 18;26:16049–. doi: 10.1038/npjpcrm.2016.49 (PMC4989925; doi:10.1038/npjpcrm.2016.49)
Supplement: Supplementary Appendix 1 [file npjpcrm201649-s1.doc]

| **APPENDIX 1. Demographics and health care resource use for asthma population prescribed LABA without ICS OR COMBI and no COPD, by number of LABA inhalers (N= 105)** | | | |
| --- | --- | --- | --- |
|  | **TOTAL N (%)** | **1 - 12 N (%)** | **13 + N (%)** |
|  | 105 | 87 (82.9) | 18 (17.1) |
| **Gender** |  |  |  |
| Female | 58 (55.2) | 51 (58.6) | 7 (38.9) |
| **Age bands** |  |  |  |
| 5-11 | 2 (1.9) | 2 (2.3) | 0 |
| 12-17 | 0 | 0 | 0 |
| 18-54 | 71 (67.6) | 61 (70.1) | 10 (55.6) |
| 55-75 | 32 (30.5) | 24 (27.6) | 8 (44.4) |
| **Ethnicity^1^** |  |  |  |
| White | 56 (53.3) | 46 (52.9) | 10 (55.6) |
| South Asian | 25 (23.8) | 19 (21.8) | 6 (33.3) |
| Black | 11 (10.5) | 10 (11.5) | 1 (5.6) |
| **Clinical measures** |  |  |  |
| Asthma Review | 92 (87.6) | 74 (85.1) | 18 (100.0) |
| Asthma Management Plan | 83 (79.1) | 66 (75.9) | 17 (94.4) |
| Asthma Step recorded | 89 (84.8) | 71 (81.6) | 18 (100.0) |
| **Asthma severity^2^** |  |  |  |
| Step 1 | 9 (10.1) | 9 (12.7) | 0 |
| Step 2 | 36 (40.5) | 30 (42.3) | 6 (33.3) |
| Step 3 | 37 (41.6) | 26 (36.6) | 11 (61.1) |
| Step 4/5 | 7 (7.9) | 6 (8.5) | 1 (5.6) |
| **Healthcare resource use** |  |  |  |
| Count IP episodes | 0 | 0 | 0 |
| IP inpatient, LABA Long-acting beta2-agonist, ICS inhaled corticosteroid, COMBI combination inhalers (ICS and Long-acting beta2-agonist), ^1^'other' and 'unknown' ethnicity categories not shown, ^2^asthma step % as a proportion of total with asthma step recorded | | | |
