# Supplementary material for: Asthma prescribing, ethnicity and risk of hospital admission: an analysis of 35,864 linked primary and secondary care records in East London
Source: NPJ Prim Care Respir Med. 2016 Aug 18;26:16049–. doi: 10.1038/npjpcrm.2016.49 (PMC4989925; doi:10.1038/npjpcrm.2016.49)
Supplement: Supplementary Appendix 2 [file npjpcrm201649-s2.doc]

| **APPENDIX 2. Characteristics of all people with one or more inpatient episode for asthma  (N=410)** | |
| --- | --- |
|  | **N (%)** |
| **Gender** | |
| Female | 245 (59.8) |
| **Age bands** | |
| 5-11 | 110 (26.8) |
| 12-17 | 36 (8.8) |
| 18-54 | 194 (47.3) |
| 55-75 | 70 (17.1) |
| **Ethnicity^1^** | |
| White | 100 (24.4) |
| South Asian | 172 (42.0) |
| Black | 81 (19.8) |
| **Clinical measures** | |
| Asthma Review | 383 (93.4) |
| Asthma Management Plan | 331 (80.7) |
| Asthma Step recorded | 357 (87.1) |
| **Asthma severity^2^** | |
| Step 1 | 46 (12.9) |
| Step 2 | 157 (44.0) |
| Step 3 | 104 (29.1) |
| Step 4/5 | 50 (14.0) |
| **SABA inhalers prescribed** | |
| 0 | 9 (2.2) |
| 1-3 | 95 (23.2) |
| 4-12 | 197 (48.0) |
| 13+ | 109 (26.6) |
| **Total ICS + COMBI inhalers prescribed** | |
| 0 | 38 (9.3) |
| 1-9 | 270 (2.3) |
| 10+ | 102 (.9) |
| **Healthcare resource use** | |
| Count IP episodes | 872 |
| IP inpatient, SABA Short-acting beta2-agonist, ICS inhaled corticosteroid, COMBI combination inhalers (ICS and Long-acting beta2-agonist), ^1^'other' and 'unknown' ethnicity categories not shown, ^2^asthma step % as a proportion of total with asthma step recorded | |
